# Manganese acquisition is facilitated by PilA in the cyanobacterium *Synechocystis* sp. PCC 6803

**DOI:** 10.1371/journal.pone.0184685

**Published:** 2017-10-10

**Authors:** Jacob J. Lamb, Martin F. Hohmann-Marriott

**Affiliations:** 1 Department of Biotechnology & PhotoSynLab, NTNU, Trondheim, Norway; 2 Department of Electronic Systems & ENERSENSE, NTNU, Trondheim, Norway; University of Hyderabad School of Life Sciences, INDIA

## Abstract

Manganese is an essential element required by cyanobacteria, as it is an essential part of the oxygen-evolving center of photosystem II. In the presence of atmospheric oxygen, manganese is present as manganese oxides, which have low solubility and consequently provide low bioavailability. It is unknown if cyanobacteria are able to utilize these manganese sources, and what mechanisms may be employed to do so. Recent evidence suggests that type IV pili in non-photosynthetic bacteria facilitate electron donation to extracellular electron acceptors, thereby enabling metal acquisition. Our present study investigates whether PilA1 (major pilin protein of type IV pili) enables the cyanobacterium *Synechocystis* PCC 6808 to access to Mn from manganese oxides. We present physiological and spectroscopic data, which indicate that the presence of PilA1 enhances the ability of cyanobacteria to grow on manganese oxides. These observations suggest a role of PilA1-containing pili in cyanobacterial manganese acquisition.

## Introduction

Cyanobacteria have fundamentally changed our planet [1]. Oxygen, which these organisms produce, complexes with transition metals, thereby limiting metal bioavailability [2–4]. One such metal is Mn, which cyanobacteria require in larger quantity than other bacteria [5], as it is an essential co-factor for the catalysis of photosynthetic water splitting. The molecular machinery for uptake of soluble Mn^2+^ has be described [6, 7], but whether and how Mn from Mn oxides can be accessed by cyanobacteria is unknown. In contrast, several biological mechanisms have been implicated for accessing iron, another oxidized transition metal, crucial for photosynthetic electron transport. There are arguments that Fe(III) that is complexed in oxides can be made available as Fe(II) by a reductive, extracellular mechanism [8, 9]. However, there remains uncertainty concerning the molecular components that are involved in reductive iron uptake.

### *Synechocystis* sp. PCC 6803 manganese acquisition

*Synechocystis* 6803 has a variety of uptake systems for elements with low bioavailability, such as iron [10–13]. An identified Mn uptake system in *Synechocystis* 6803 consists of is an ATP-binding membrane transporter encoded by the genes *sll1598-1600* [6]. This manganese uptake system is specific for soluble Mn(II), but no Mn uptake system for other oxidized Mn states have been identified. Interactions of Mn uptake with the Fur (Ferric Uptake Regulator) uptake pathway have been observed, suggesting that Mn and Fe uptake are coordinated [14, 15], or that it is difficult for cyanobacteria to distinguish between Fe and Mn.

Type IV pili of non-photosynthetic soil bacteria have been implicated for mediating extracellular reduction of metal oxides. Observations using conducting-probe atomic force microscopy [16], and scanning tunneling microscopy [17], demonstrated that these thick pili structures are electrically conductive, coining the name ‘bacterial nanowires’. These pili are composed of pilins that form extracellular protein fibers and thought to facilitate electron donation to external electron acceptors, such as metallo-oxides, thus facilitating respiration in anaerobic conditions [16, 17]. Contrary to this, *Shewanella oneidensis* can form *tubular* extracellular extensions in the absence of pilins. In a study be Pirbadian and colleagues [18] extracellular extensions were observed in a strain in which the main pilin (pilA) had been genetically deleted. Imaging and selective staining was used shown that the extracellular extensions are evaginations of the outer membrane extensions and house multi-heme cytochromes [18]. In addition to sustaining respiration, another—so far untested—consequence of reducing metallo-oxides is that this process may also unlock manganese that is essential for cyanobacterial growth.

Kranzler et al [9] have presented evidence that *Synechocystis* sp. PCC 6803 is capable of acquiring Fe through a reductive two-step process. First reduction acts as an activation step, followed by the transport of the now reduced Fe(II) through the plasma membrane. It is proposed that reductive activation step occurs outside of the cell, on the surface of the outer membrane or in the periplasmic space [8, 9]. That oxidized Fe can be utilized as an iron source by *Synechocystis* sp. PCC 6803 is consistent with growth experiments on various oxidized iron sources [19]. This 'reductive activation' may provide a mechanism for assessing a great variety of metallo-oxide conjugates, as well as metal chelator complexes cyanobacteria may encounter in nature. Recently, the electron transport by pili has also been suggested for accumulation of arsenic, iron and manganese-containing particles in proximity to pili [20]. However, the deposition of these particles would likely involve the oxidation of extra-cellular metals and metalloids by Pili.

### Manganese-stress response in *Synechocystis* sp. PCC 6803

During Mn-limited growth conditions, the photosynthetic machinery of *Synechocystis* sp. PCC 6803 undergoes a dynamic change [21]. When *Synechocystis* 6803 experiences Mn limitation, an increased 77 K fluorescence emissions at 682 nm, as well as lowered oxygen evolution rates due to the accumulation of unassembled PSII, have been reported [21]. The increase in chlorophyll fluorescence emission at 682 nm coincides with a decrease in 720 nm emission that is largely attributed to PSI fluorescence. This reduced chlorophyll fluorescence emission is ascribed to a decrease in PSI core proteins and the dynamic organizational changes from PSI trimers to monomers.

### Pili in *Synechocystis* sp. PCC 6803

Many cyanobacterial genomes contain genes that are part of the type IV protein export machinery, which processes pili components. The number of *pilA* pilin genes, coding for the main component of pili, varies between different cyanobacteria, and it is thought that this diversity may be due to the fine-tuning of functional characteristics. The genome of *Synechocystis* sp. PCC 6803 contains 10 putative *pilA* pilin genes, organized in three polycistronic operons (*sll1694-1695*; *slr1928-slr1931*; *slr2015-slr2017*) and one monocystronic operon (*slr1456*). The operon comprising of the genes *sll1694-1695* (*pilA1* and *pilA2*, respectively), is critical for construction of extracellular pili. This is because the protein encoded by *sll1694* (*pilA1*) is the principle pilin protein of the extracellular Type IV pili [22–24]. Recently it was suggested that in *Shewanella oneidensis*, these extracellular appendixes are outer membrane extensions that house many multi-heme cytochromes, and not pilin-based [18]; however, this has not been observed to be the case in *Synechocystis* sp. PCC 6803. The protein encoded by *sll1695* is another *pilA* pilin that has been suggested to be confined to the cytoplasmic side of the cytoplasmic film [22, 23]. Dissimilar to the first *Synechocystis* 6803 strain isolated, a glucose tolerant lab strain utilized by many research groups, including this study, has lost its motility capacity because of a mutation in the SpkA signaling kinase [25, 26]; however, the biogenesis pili is not influenced by this mutation.

### Aim of this study

In this report, we investigate if PilA1 of the cyanobacterium *Synechocystis* sp. PCC 6803 are involved in the acquisition of Mn from Mn oxides.

## Results

**Fig 1:**
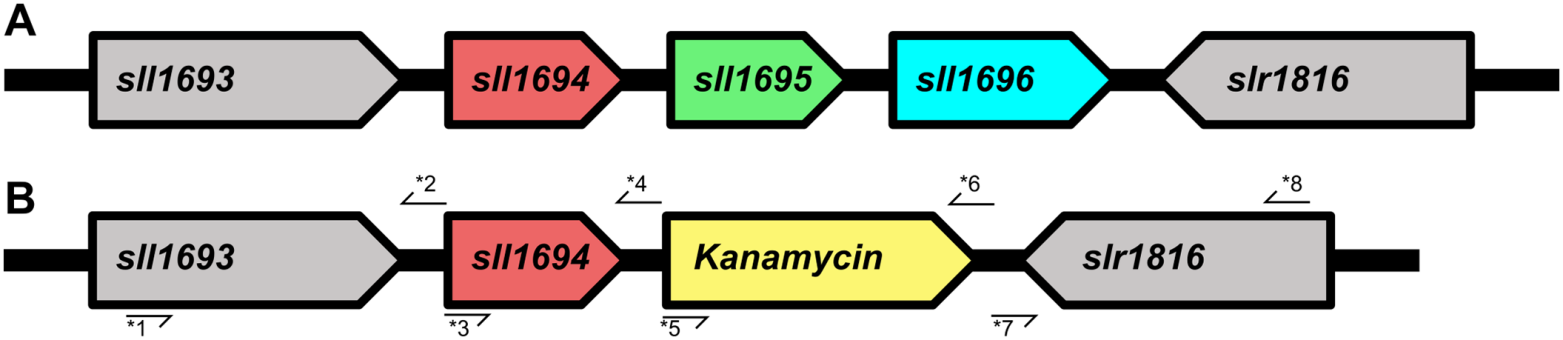
*Synechocystis* sp. PCC 6803 operon containing *pilA1* deletion schematic. The genomic organization of the *pilA1*-containing operon housed between *sll1693* and *slr1816* in the native wild type. The relative positions of primers used for strain construction are shown, with the number corresponding to the specific primer in Table 1.

To investigate the possible role of pili in the acquisition of Mn, we utilized the same PilA-deficient mutant (Δ*sll1694*) that was previously employed to investigate the role of pili in Fe acquisition ([19]; Fig 1; Table 1).

**Table 1:** Gibson assembly primers for Δ*sll1694* strain generation.

Primer name	Primer sequence
Left_fwd (*1)	GGAAACAGCTATGACCATGGGATTGGCGGCAGTTATTTAAGG
Left_rev (*2)	ACACAACGTGCTCTATGCGGCCGCTGATTGTCTTCTTCCTTCTGTAGGG
sll1694_fwd (*3)	ACAGAAGGAAGAAGACAATCATGGCTAGTAATTTTAAATTCAAACTCC
sll1694_rev (*4)	TTGAGACACAACGTGTGCGGCCGCTCATAACAATAGTGTGAAAATATTAACCC
Kan_fwd (*5)	AGGAAGAAGACAATCAGCGGCCGCACACGTTGTGTCTCAAAATCTC
Kan_rev (*6)	TTAAAATTATCAATCAGCGGCCGCATACAACCAATTAACCAATTCTG
Right_fwd (*7)	TGGTTAATTGGTTGTATGCGGCCGCTGATTGATAATTTTAATCTAGATCTCCATTC
Right_rev (*8)	GGTTTTCCCAGTCACGACCAGAATAAGATCAAATCGAACCCCAA

*[number], refers to relative position of specific primer in Fig 1.

These studies revealed that with medium containing ferric ammonium citrate and MnCl_2_ as Fe and Mn sources, respectively, wild type and the Δ*sll1694* mutant have almost identical spectral and growth characteristics in liquid medium. However, the Δ*sll1694* mutants grow with longer doubling times, and to lower densities on plates, as well as exhibiting different spectral characteristics. To investigate the role of pili in Mn acquisition, we therefore decided to initially investigate growth on different Mn sources on plates. The purest commercially available forms of all the required chemicals for making BG11 were purchased for use in all experiments.

### Growth analysis

**Fig 2:**
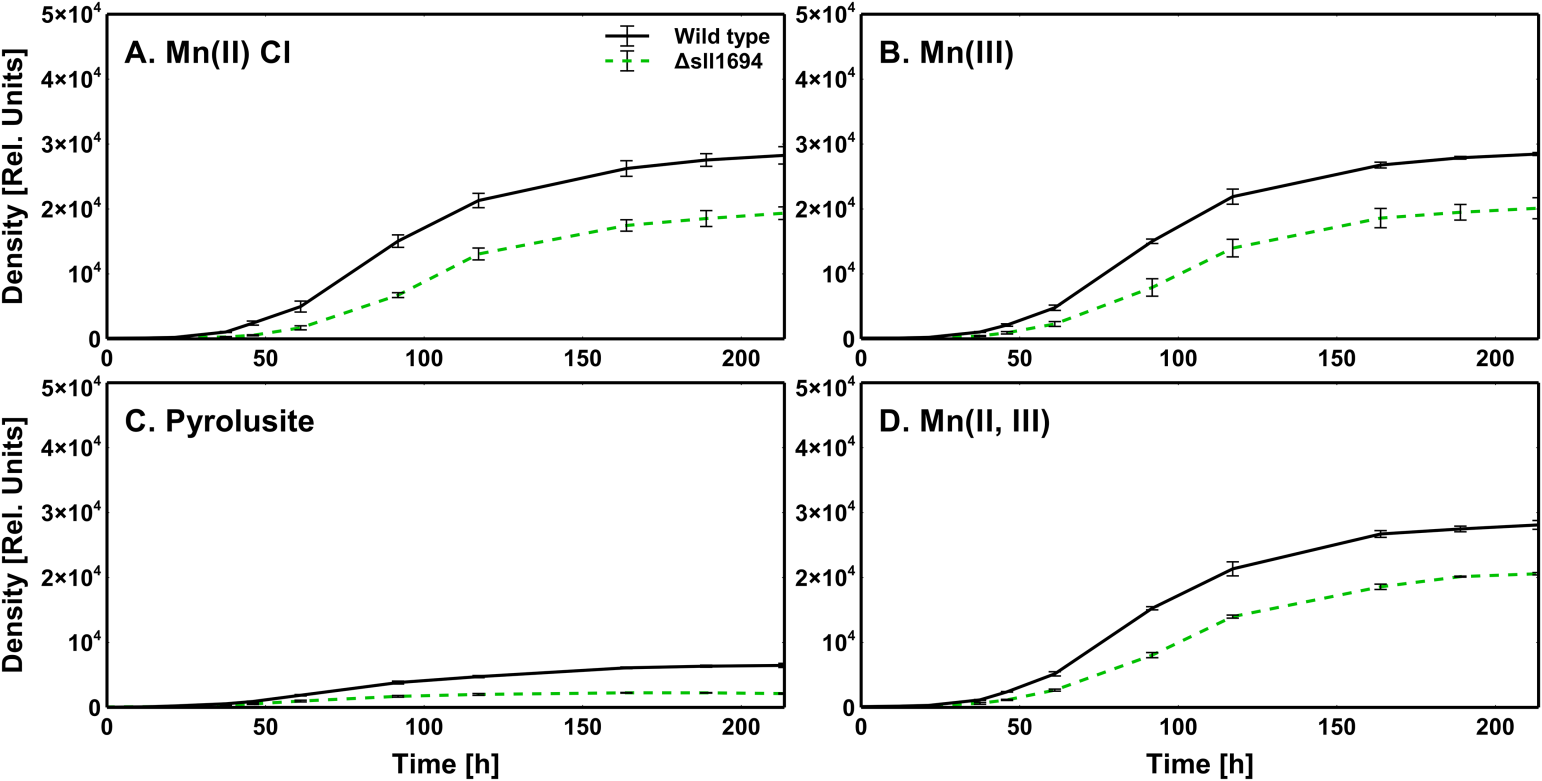
Growth of various BG11 media. Photoautotrophic growth characteristics of wild type and the Δ*sll1694* strain grown on agar plates containing BG11 with Mn(II) chloride (A), Mn(III) (B), pyrolusite (C), and Mn(II, III) (D) as the exclusive manganese sources (Table 2). The exponential doubling time of these growth conditions for both wild type and the Δ*sll1694* strain are shown in Table 3. Trend shown is indicative of nine separate measurements (three strains with three replicates each). Error bars showing the standard error of the biological replicates.

As the photosynthetic machinery requires Mn, we hypothesized that autotrophic growth will be impeded if Mn bioavailability is limited. Maximum growth densities of strains grown on agar plates that contained a variety of exclusive Mn sources (Table 2) were assessed. For wild type and the Δ*sll1694* mutant, autotrophic growth on BG11 media with pyrolusite as exclusive source of Mn was decreased compared to growth on BG11 media with Mn(II), Mn(III), or Mn(II, III) as the exclusive Mn source (Fig 2; Tables 2 & 3). Moreover, in all conditions, the Δ*sll1694* strain exhibited lower growth densities than the wild type.

**Table 2:** BG11 media variants for analysis.

Exclusive Mn source	BG11 variant short name			
	Mn(II) Cl	Mn(III)	Pyrolusite	Mn(II,III)
Manganese(II) chloride	+	-	-	-
Manganese(III) oxide	-	+	-	-
Manganese(IV) oxide	-	-	+	-
Manganese(II, III) oxide	-	-	-	+

**Table 3:** Exponential growth doubling times and maximal growth densities of *Synechocystis* sp. PCC 6803 in various growth conditions.

	Growth doubling times (h)		Maximal growth density (relative units)	
BG11 variant	Wild type	Δ*sll1694*	Wild type	Δ*sll1694*
Mn(II) Cl	22.23 ± 1.00	24.89 ± 1.25	28260 ± 1336	19355 ± 971
Mn(III)	22.32 ± 1.18	24.82 ± 1.19	28469 ± 228	22120 ± 1619
Pyrolusite	30.80 ± 1.52	34.78 ± 1.32	6465 ± 319	2128 ± 81
Mn(II,III)	23.15 ± 0.56	28.43 ± 1.24	28104 ± 680	20582 ± 179

±, standard deviation of biological replicates

**Fig 3:**
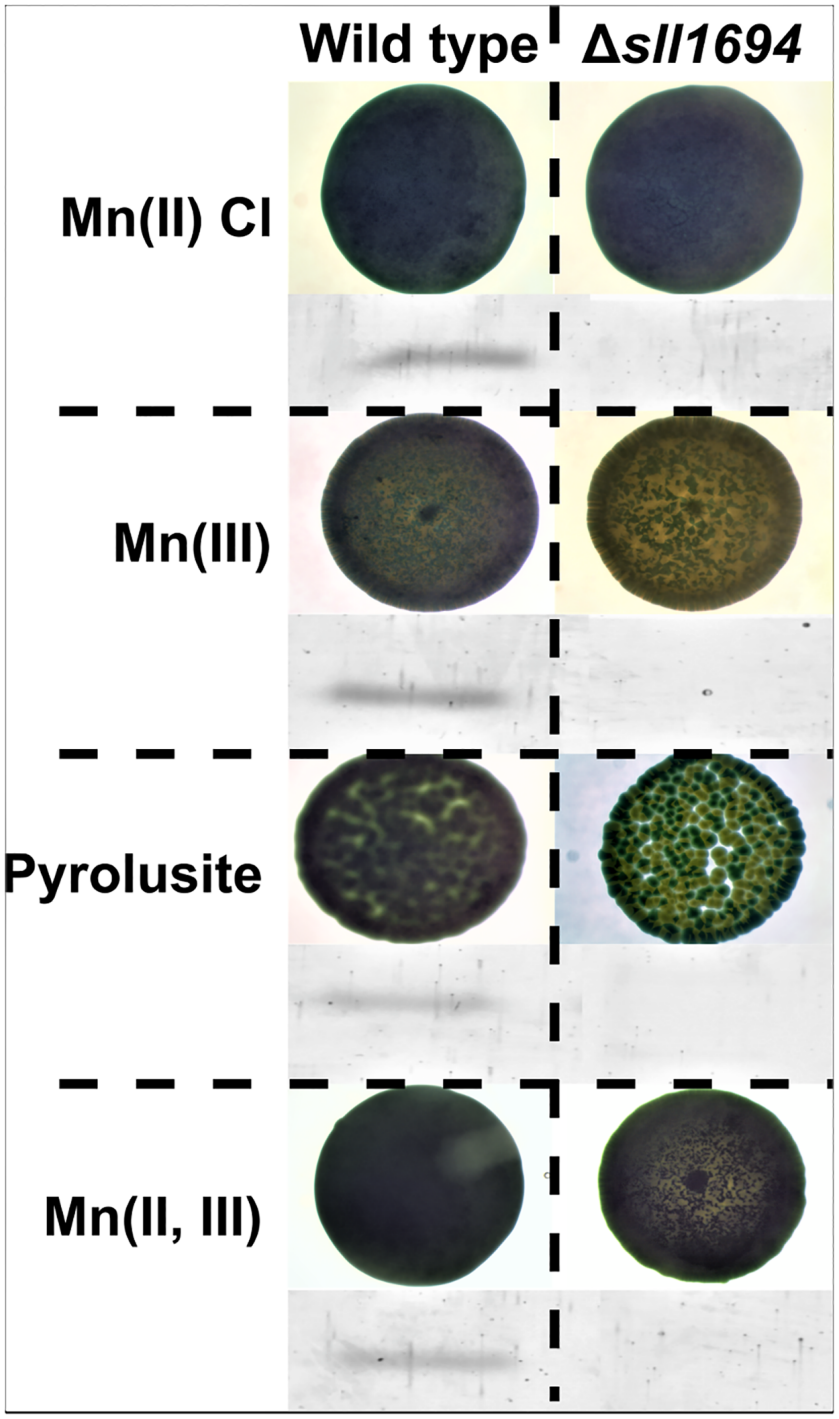
*Synechocystis* sp. PCC 6803 growth phenotype observed on agar plate. Images of wild type and Δ*sll1694* strains on petri dishes containing agar-solidified BG11 medium with Mn(II) chloride (A), Mn(III) (B), pyrolusite (C), and Mn(II, III) (D) as the exclusive manganese sources. The extracellular protein harvested from the strains and normalized to chlorophyll content before analyzed by gel electrophoresis. The PilA protein (encoded by *sll1694*) is present in wild type strain where *sll1694* is intact, but not in the Δ*sll1694* strain where *sll1694* has been interrupted.

The Δ*sll1694* strain cells that did not produce the PilA protein exhibited a dramatic change in coloration in all growth conditions, when compared to wild type used in this study (Fig 3). This phenotype is exaggerated when grown on BG11-containing pyrolusite as the sole manganese source. The discoloration may be indicative of the statistical nature of having access to metallic oxide particles where areas of low access reduce photosynthetic machinery due to reduced metal bioavailability.

### Oxygen evolution

**Fig 4:**
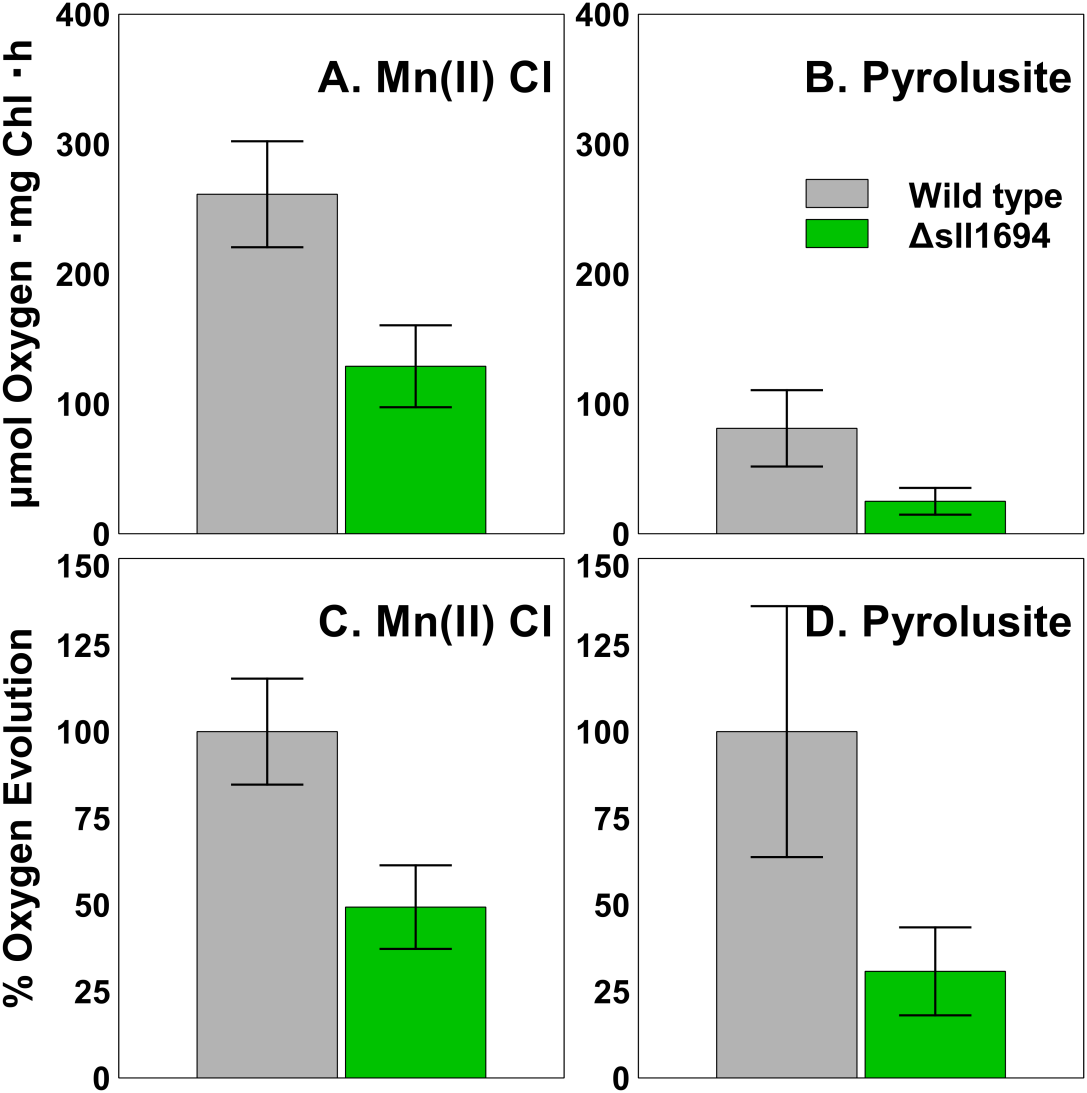
Oxygen evolution of *Synechocystis* sp. PCC 6803 grown on agar plates. The wild type and Δ*sll1694* strain were grown using BG11 agar media with Mn(II) chloride (A & C) or pyrolusite (B &D) as the exclusive Mn source. The oxygen evolution was measured in μmol O_2_・mg Chl^-1^・h^-1^ (A & B), and as a percentage of the wild type oxygen evolution (C & D). Trend shown is indicative of nine separate measurements (three strains with three replicates each). Error bars showing the standard error of the biological replicates.

The activity of PSII was assessed directly by measuring oxygen evolution with a Clark-type electrode (Hansatech, UK). These measurements revealed that oxygen evolution is not different in between wild type cells grown on MnCl_2_ or pyrolusite as exclusive source of Mn. However, for the Δ*sll1694* strain, oxygen evolution was roughly 50% that of the wild type (Fig 4), when cells were grown on BG11 that included MnCl_2_. Oxygen evolution was further reduced when pyrolusite was used as the manganese source, where the oxygen evolution of the Δ*sll1694* strain was roughly 30% of the wild type. The pattern of oxygen evolution are consistent with the reduction in photosynthetically-driven growth, due to limited access to manganese.

### Whole cell absorption spectra

The light absorption spectra of *Synechocystis* sp. PCC 6803 whole cells provide an insight into the status of the photosynthetic machinery. When *Synechocystis* sp. PCC 6803 grows in conditions of low/no photosynthetic machinery stress, the majority of the chlorophyll *a* is associated with photosystem I (PSI) and photosystem II (PSII), exhibiting major absorption bands around both 435 nm (Soret band) and 680 nm. The phycobillisomes (containing phycocyanin and allophycocyanin), result in a composite absorption band around 625 nm that is associated with mainly associated with PSII. A chlorophyll *a* / phycobilin ratio can be determined by calculating the ratio of the 625 nm to 680 nm absorption bands.

The amount of light-harvesting pigments that are present can be used to deduce the status of the photosynthetic machinery. A relatively high phycobilisome to chlorophyll *a* ratio is indicative of active electron generation by PSII (generally seen in non-stress conditions). However, when the number of phycobilisomes to chlorophyll *a* is reduced, a reduced rate of active electron generation in PSII is present (generally seen in light-stress conditions).

**Fig 5:**
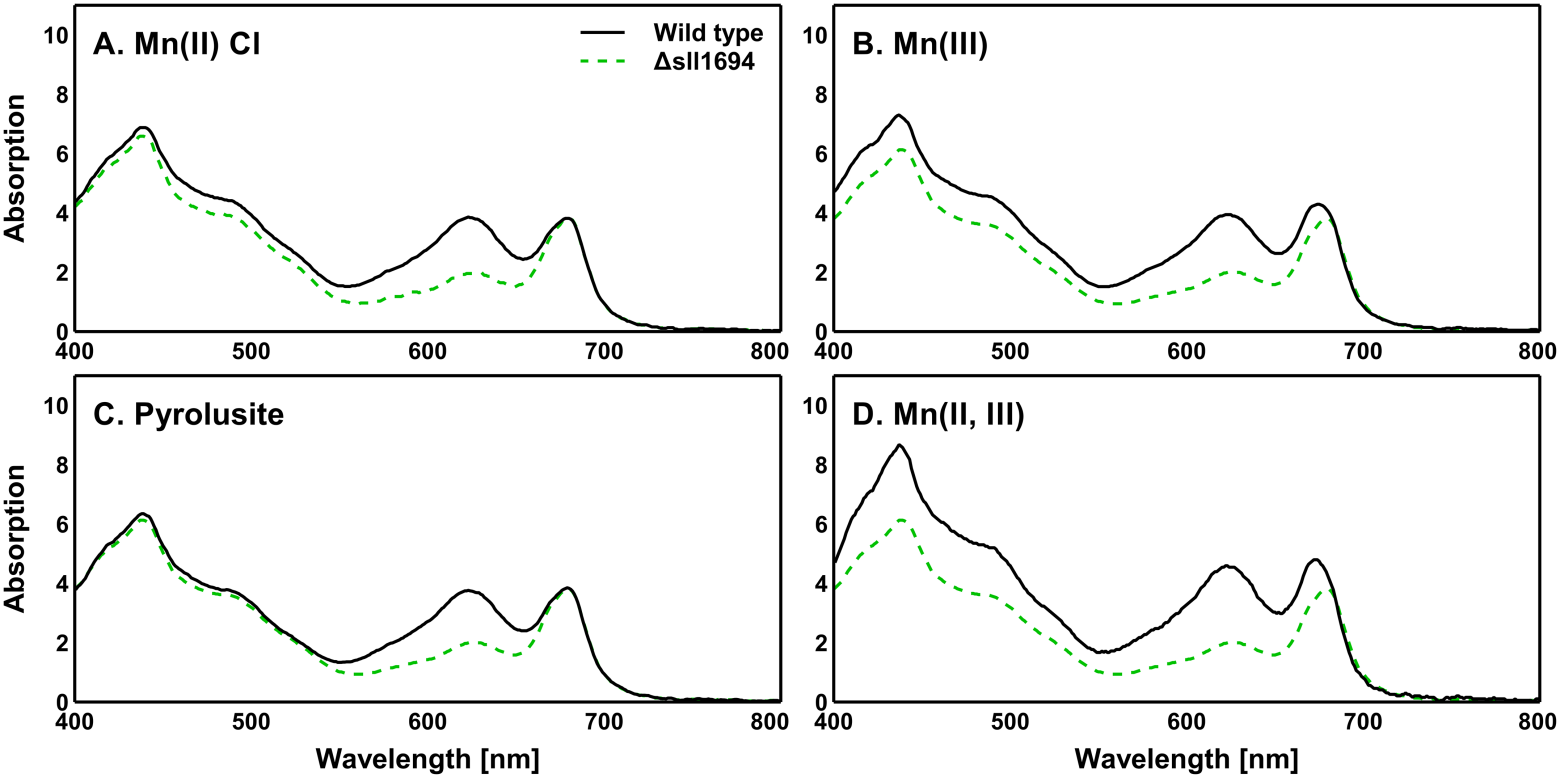
Absorption spectra of a photoautotrophically grown *Synechocystis*. sp. PCC 6803 agar plate cultures. Wild type and Δ*sll1694* strains were grown on agar plates containing BG11 with Mn(II) chloride (A), Mn(III) (B), pyrolusite (C), and Mn(II, III) (D) as the exclusive manganese sources (Table 2). Samples were standardized to an OD750 of 0.3, then traces normalized to 700 nm. Trend shown is indicative of nine separate measurements (three strains with three replicates each). The standard error of these replicates was calculated. Wild type standard error: ±3.9×10^−2^ (A), ±4.1×10^−2^ (B), ±2.8×10^−2^ (C), ±4.5×10^−2^ (D). Δ*sll1694* standard error: ±3.2×10^−2^ (A), ±5.1×10^−2^ (B), ±4.2×10^−2^ (C), ±5.2×10^−2^ (D).

The Δ*sll1694* strain had a severely reduced phycobilin to chlorophyll ratio irrespective of growth condition, whereas, the wild type strain showed no significant change in this ratio irrespective of growth condition (Fig 5; Table 2). The cause of this could be due to the reduced growth rate of the mutant strain, resulting in the lower growth densities observed, exposing the mutant strain cells to higher light levels than the dense wild type cells, requiring a down-regulation in light harvesting.

Decrease in growth rate and oxygen evolution of the Δ*sll1694* mutant grown on pyrolusite, a source of Mn that is not considered to be bioavailable, indicates that Mn availability may be limited.

### Chlorophyll fluorescence characteristics at 77K

**Fig 6:**
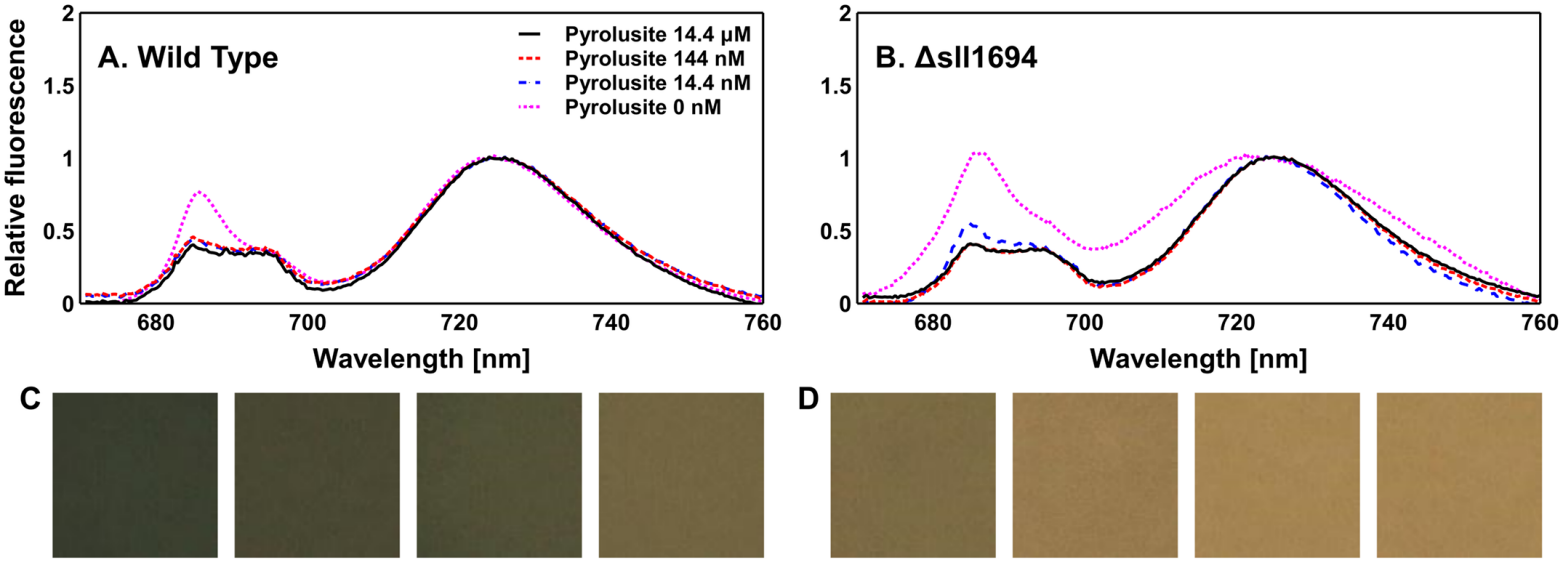
*Synechocystis* sp. PCC 6803 77 K fluorescence emission as a function of pyrolusite concentration. 77 K fluorescence emission characteristics of the wild type (A) and the Δ*sll1694* (B) strain were obtained in response to varying pyrolusite concentrations. Images representing the growth characteristics of the wild type (C) and the Δ*sll1694* (D) strain were also acquired in response to the varying pyrolusite concentrations. Concentration series were 14.4 μM, 144 nM, 14.4 nM and 0 nM of pyrolusite. The samples were excited by a 440 nm LED light source. Traces were normalized to the PS I peak at 725 nm. Trend shown is indicative of three separate biological replicates. The standard error of these replicates was calculated. Wild type standard error: ±2.7×10^−2^ (14.4 μM pyrolusite), ±3.1×10^−2^ (144 nM pyrolusite), ±3.3×10^−2^ (14.4 nM pyrolusite), ±3.8×10^−2^ (0 nM pyrolusite). Δ*sll1694* standard error: ±3.6×10^−2^ (14.4 μM pyrolusite), ±5.6×10^−2^ (144 nM pyrolusite), ±6.2×10^−2^ (14.4 nM pyrolusite), ±6.9×10^−2^ (0 nM pyrolusite).

Chlorophyll fluorescence emission spectra at 77K are a very sensitive method for detecting Mn depletion in cyanobacteria. Mn-limited cyanobacteria exhibit an increased fluorescence emission at 682 nm [21]. This has been suggested to be due to the accumulation of unassembled PSII [21], or could also be the result of a reduction in the number of PSI per cell. In order to detect the possible presence of a Mn-deplete phenotype, an assay was designed in which pyrolusite concentrations in the growth medium were systematically altered. The *sll1694* deletion mutant requires higher pyrolusite concentrations than the wild type to avoid increased fluorescence emission at 682 nm (Fig 6). The wild type can apparently access Mn from pyrolusite at the lowest concentration tested, while 77K fluorescence spectra clearly exhibit signs of Mn-depletion stress in the Δ*sll1694* strain. That the wild type is able to access Mn even when no source of Mn is intentionally added can be gleamed from 77K fluorescence spectra (Fig 6). This indicates that some Mn is present originating from the stored Mn within the inoculating cells, as well as potential contamination from other constituents of the BG11 medium. Since trying to chelate Mn will likely also effect other trace metal ions, adding uncertainty about the growth medium and making our study not comparable with the body of existing literature that utilize standard BG11 media. The fluorescence emission spectra from colonies for all strains grown on standard BG11 (MnCl_2_ and Ferric ammonium citrate, as Mn and Fe sources respectively) were similar for the wild type and the Δ*sll1694* strain.

## Discussion

Mn is a critical component of the photosynthetic apparatus of cyanobacteria. In the presence of oxygen, Mn forms insoluble oxides that are not bioavailable. Consequently, Mn can be a growth-limiting nutrient for cyanobacteria in natural environments.

We investigated, if bacterial pili play a role in making oxidized manganese minerals available and discuss the physiological observations. In strains in which the *pilA1* gene (*sll1694*) is genetically interrupted, no PilA protein are formed and consequently no pilins are formed. The interruption of *sll1694* likely also interrupts the expression of two additional genes that are present in the same polycistronic operon, *pilA2* (*sll1695*), which encodes another pilin PilA2, and *sll1696*, which codes for a hypothetical protein with unknown function.

Deletion of *sll1694* has a serve phenotypical consequence for growth on agar plates [19]. Growth on plates is diminished when the Δ*sll1694* strain is grown on the manganese source MnCl_2_, which appears readily available in liquid medium. We have shown previously that this phenotype is likely due the inability to utilize oxidized sources of iron [19], and not limited by Mn availability. When the standard Mn source (MnCl_2_) is replaced by manganese oxides, the wild type cells grow faster than the *pilA1*-deficient strain. Pili-mediated motility is not a reason for the observed differences in growth, as the *Synechocystis* strain used in our study has lost mobility due to a mutation in the SpkA signaling kinase [25, 26]. The growth experiments are thus consistent with type IV pili having a role in manganese acquisition.

As manganese is a critical element for PSII-mediated water splitting, physiological and spectral changes in PSII can be evaluated as a reporter for the cellular availability of Mn. Oxygen evolution measurements reveal that cells that lack PiliA1 produced about half the oxygen of wild type cells. Fluorescence emission spectra at 77K point to unassembled PSII in cells that lack *sll1694*, but this could also be due to a reduction in the number PSI, or unconnected CP43’ antenna. Furthermore, diminished amounts of phycobillins in Δ*sll1694* cells indicates that cells are stressed. This could be due to Mn-related stress, or could be an artifact of the reduced growth-rate observed in the stain lacking the *sll1694* gene. Together, these data suggest diminished and faulty assembly of PSII, a reduction in PSI, or the accumulation of unconnected CP43’ antenna, due to a lack of bioavailable Mn in the Δ*sll164* strain. Recent studies by Linhartová et al [27] showed in a prepillin peptidase-deficient strain (lacking the *pilD* gene, *slr1120*) that an accumulation of unprocessed PilA1 protein within the cell leads to decrease of assembled PSII. Interestingly, a role of *sll1694* for the incorporation of chlorophylls into light harvesting complexes and photosystems has been postulated based on deletion of *sll1694* under certain conditions [28]. The underlying reason for this phenotype may be insufficient supply of Mn.

Pili may aide in Mn acquisition by providing a reductive mechanism for accessing Mn oxides, analogous to the mechanism suggested by our group [19] for gaining access to iron locked within iron oxides. We hypothesis that the wild type strain can use the cell membrane-based Mn^**2+**^ uptake system as well as the extracellular pili to access Mn^3+^ and Mn^4+^ within iron oxides. The PilA-deficient mutant, however, has only the cell membrane Mn uptake system available, and thus only soluble Mn^2+^ can be accessed by this mutant. Other metal oxides have also been shown to serve as electron acceptors for some non-photosynthetic soil bacteria [16, 17], to enable respiration in anaerobic conditions. There is also independent evidence for a reductive iron uptake mechanism in cyanobacteria [9]. That this reduction mechanism involves pili is consistent with reports of iron acquisition in the model cyanobacterial system *Synechocystis* sp. PCC 6803 [19].

## Conclusion

The results of study demonstrate that pili aide in the acquisition of Mn from Mn oxides, but more detailed insights on the electron transport between the cell and extracellular electron acceptors, such as Mn oxides, is required to clearly define the role of pili in this process. It is not surprising that the organisms responsible for our oxygen-rich environment have adapted and evolved mechanisms to improve bioavailability of the metals that are essential for photosynthesis.

## Materials and methods

### Growth and maintenance of stock cultures

Cells were maintained on BG11 [29] agar plates containing 5 mM glucose, 20 μM atrazine and appropriate antibiotics where applicable [30], whereas experimental cultures contained no glucose, no atrazine, and no antibiotics (grown phototrophically). Liquid cultures were established in 300 mL Erlenmeyer flasks that have been specifically modified as described by Eaton-Rye [30]. Both plates and liquid cultures were grown under constant illumination (30 μE.m^-2^.s^-1^), at 30°C. Liquid cultures were provided with filtered aeration via small aquarium pumps. The air was additionally bubbled through deionized water (18 MΩ) to prevent dehydration of cell cultures.

### Alternative manganese sources

BG11 media without manganese(II) chloride was supplemented with either 1.11 μg.mL^-1^ (14.4 μM Mn) of manganese(III) oxide, 1.22 μg.mL^-1^ (14.4 μM Mn) of manganese(IV) oxide, or 1.07 μg.mL^-1^ (14.4 μM Mn) of manganese(II,III) oxide (Table 2).

### Polymerase chain reaction

Primers for PCR analysis were ordered as needed in the 5’ to 3’ orientation with specific restriction enzyme cut sites upstream of the 5’ end (Table 1), through Sigma-Aldrich (Sigma-Aldrich, UK).

The reactions were performed in 1x Phusion amplification buffer containing 0.4 mM of each dNTP (dATP, dCTP, dGTP and dTTP), 0.4 mM of each primer and 0.05 units/μL Phusion polymerase (Thermo Fisher Scientific, USA). PCR conditions were: (1) initial denaturing step at 98°C for 30 s; (2) 14 cycles of 98°C for 7 s, annealing at 62°C (-1°C per cycle) for 20 s, extension at 72°C for 30 s/kb; (3) 16 cycles of 98°C for 7 s, annealing at 62°C for 20 s, extension at 72°C for 30 s/kb; then (4) final extension at 72°C for 5 min. PCR products were cleaned using a QIAquick PCR purification kit (Qiagen, Duesseldorf, Germany).

### Separation of DNA samples by gel electrophoresis

DNA samples were separated by electrophoresis using 0.8% agarose gels in the presence of 10 mM NaOH and 73 mM boric acid at pH 8.0. The running condition was 70 V for 45 min at room temperature. Prior to loading, samples were mixed with 10× loading buffer (0.25% bromophenol blue, 0.25% xylene cyanol FF and 30% glycerol). DNA was visualized by exposure to a UV light. Gel images were captured using a GelDoc (BioRad, USA).

### Gibson plasmid construction

Gibson assembly was performed using the Gibson Assembly Master Mix (NEB, USA). The reaction was carried out using 100 ng of vector DNA with 3-fold molar excess of insert fragments. The DNA volume was adjusted with deionized water (18 MΩ) to 10 μL. An equal amount (10 μL) of Gibson Master Mix was added to the DNA fragments. The reaction mix was then incubated at 50°C for 60 minutes. Once reaction was complete, the sample was transformed into DH5α competent cells.

### Transformation of *Synechocystis* sp. PCC 6803

Liquid cultures were grown in the presence of glucose to an OD of approximately 0.5. Cells were then centrifuged at 2760*g* for 10 min and suspended in 0.5 mL BG-11 media to a final OD of 2.5 in a sterile tube. Approximately 5 μg of plasmid DNA was added to tubes and incubated at 30°C under 30 μE.m^-2^.s^-1^ of illumination for 6 h with gentle shaking at the 3 h mark [31]. Negative controls with no DNA were also included. Samples were spread over sterile nitrocellulose paper on BG-11 plates supplemented with glucose and incubated overnight. The filters, with cells attached, were then transferred to plates containing glucose, atrazine and appropriate antibiotics. After two weeks, single colonies were picked and streaked out weekly for three weeks to promote complete segregation. Colony PCR was used to verify complete segregation using primers in Table 1.

### Solid-media growth curve

Liquid cultures of *Synechocystis* sp. PCC 6803 were grown in the presence of 5 mM glucose to an OD of 1.0 using a custom built spectrophotometer [32]. Cells were centrifuged at 2760 g for 10 min at 25°C followed by three washing steps in BG11 without Mn(II) Cl and resuspended to an OD of 1.0. A small amount of this liquid culture was used to inoculate 2 mL of BG11 without Mn(II) Cl to an OD of 0.1. Two micro liters of each of the culture samples were spotted onto specific BG11 agar growth plates with appropriate growth conditions (specific manganese sources, Table 2). Cultures were maintained under constant temperature (30°C) and illumination (30 μE.m^-2^.s^-1^). Raw images of each culture were taken at specific intervals over a 200 h period using a custom-built plate imager. The integrated intensity of the cultures was then compared to the agar background to give a relative growth parameter.

### Growth characteristics

Liquid cultures of *Synechocystis* sp. PCC 6803 were grown in the presence of 5 mM glucose to an OD of 1.0 using a custom built spectrophotometer [32]. Cells were centrifuged at 2760 g for 10 min at 25°C followed by three washing steps in BG11 without Mn(II) Cl and resuspended to an OD of 1.0. Two micro liters of the culture samples were spotted onto specific BG11 agar growth plates with a concentration series of14.4 μM, 144 nM, 14.4 nM and 0 nM of pyrolusite. Cultures were maintained under constant temperature (30°C) and illumination (30 μE.m^-2^.s^-1^). Pictures were taken after 10 days of growth to be used as a proxy for their final growth density.

### Sodium dodecyl sulphate-polyacrylamide gel electrophoresis

In this study, 12% SDS-PAGE using a Tris-glycine buffer system was used. The running condition was 200 V for 55 min at room temperature. Directly after completion of electrophoresis, the gels were removed from the glass cassette and soaked in a gel-fixing solution (50% (v/v) ethanol and 10% (v/v) acetic acid in ddH2O) for 1 h. The gel was then incubated at room temperature overnight with slight agitation in gel-washing solution (50% (v/v) methanol and 10% (v/v) acetic acid in ddH2O). For visualization of protein bands the gel was covered with Coomassie stain (0.1% (w/v) Coomassie blue R350, 20% (v/v) methanol and 10% (v/v) acetic acid in ddH2O) for 3 to 4 h with slight agitation. Destain solution (50% (v/v) methanol and 10% (v/v) acetic acid in ddH2O) was then used several times to remove excess Coomassie stain. The gel was then stored indefinitely in a storage solution (5% (v/v) acetic acid in ddH_2_O).

### Whole cell absorption spectra

Whole cell absorption spectra were measured using a U-3010 Hitachi (Japan) spectrophotometer with integrating sphere. Cells were suspended in BG11 containing HEPES-NaOH, pH 7.5, to an OD_750_ of 0.3.

### 77 K fluorescence

Cells were diluted with BG-11 containing HEPES/NaOH, pH 7.5, to a chlorophyll concentration of 2 μg.mL^-1^ measured using a custom built fluorometer [33]. Cells were then transferred into glass tubes, frozen in liquid nitrogen and fluorescence emission assessed using a custom built fluorescence spectrometer [34]. Traces were normalized to a PSI peak at 725 nm in the emission spectra.

### Oxygen evolution of whole cells

*Synechocystis* sp. PCC 6803 cells were obtained from specific BG11 growth plates and resuspended in BG11 with the correct Mn source corresponding to their specific BG11 growth condition. Cells were centrifuged at 2500g for 8 min at 25°C and washed twice in their specific BG11 containing 25 mM Hepes/NaOH, pH 7.5 and diluted to a chlorophyll concentration of 10 μg mL^-1^. A Clark-type electrode (Hansatech, UK) was used for measurements. A volume of 1 mL was transferred into the electrode reaction chamber with constant stirring. Saturating light was provided by a multicolour PAM (Waltz, Germany).
